# webTWAS: a resource for disease candidate susceptibility genes identified by transcriptome-wide association study

**DOI:** 10.1093/nar/gkab957

**Published:** 2021-10-20

**Authors:** Chen Cao, Jianhua Wang, Devin Kwok, Feifei Cui, Zilong Zhang, Da Zhao, Mulin Jun Li, Quan Zou

**Affiliations:** Yangtze Delta Region Institute (Quzhou), University of Electronic Science and Technology of China, Quzhou, China; Institute of Fundamental and Frontier Sciences, University of Electronic Science and Technology of China, Chengdu, China; Department of Biochemistry & Molecular Biology, Alberta Children's Hospital Research Institute, University of Calgary, Calgary, Canada; Department of Pharmacology, School of Basic Medical Sciences, Tianjin Medical University, Tianjin, China; School of Computer Science, McGill University, Montreal, Canada; Yangtze Delta Region Institute (Quzhou), University of Electronic Science and Technology of China, Quzhou, China; Institute of Fundamental and Frontier Sciences, University of Electronic Science and Technology of China, Chengdu, China; Yangtze Delta Region Institute (Quzhou), University of Electronic Science and Technology of China, Quzhou, China; Institute of Fundamental and Frontier Sciences, University of Electronic Science and Technology of China, Chengdu, China; Yangtze Delta Region Institute (Quzhou), University of Electronic Science and Technology of China, Quzhou, China; Institute of Fundamental and Frontier Sciences, University of Electronic Science and Technology of China, Chengdu, China; Department of Pharmacology, School of Basic Medical Sciences, Tianjin Medical University, Tianjin, China; Yangtze Delta Region Institute (Quzhou), University of Electronic Science and Technology of China, Quzhou, China; Institute of Fundamental and Frontier Sciences, University of Electronic Science and Technology of China, Chengdu, China

## Abstract

The development of transcriptome-wide association studies (TWAS) has enabled researchers to better identify and interpret causal genes in many diseases. However, there are currently no resources providing a comprehensive listing of gene-disease associations discovered by TWAS from published GWAS summary statistics. TWAS analyses are also difficult to conduct due to the complexity of TWAS software pipelines. To address these issues, we introduce a new resource called webTWAS, which integrates a database of the most comprehensive disease GWAS datasets currently available with credible sets of potential causal genes identified by multiple TWAS software packages. Specifically, a total of 235 064 gene-diseases associations for a wide range of human diseases are prioritized from 1298 high-quality downloadable European GWAS summary statistics. Associations are calculated with seven different statistical models based on three popular and representative TWAS software packages. Users can explore associations at the gene or disease level, and easily search for related studies or diseases using the MeSH disease tree. Since the effects of diseases are highly tissue-specific, webTWAS applies tissue-specific enrichment analysis to identify significant tissues. A user-friendly web server is also available to run custom TWAS analyses on user-provided GWAS summary statistics data. webTWAS is freely available at http://www.webtwas.net.

## INTRODUCTION

First proposed by Gamazon *et al.* (1) in 2015, the transcriptome-wide association study (TWAS) has emerged as a powerful method for investigating associations between genetic variants and disease or disease-related complex traits. TWAS utilizes a reference panel of genotype and expression quantitative trait data (such as GTEx (2)) to fit a regression model predicting a target gene's expression from genotype. This model is used to impute the genetically regulated expression (GReX) of the gene from genotype data in a genome-wide association study (GWAS). The imputed GReX values are then used to discover associations between the target gene and the phenotype of interest. While genome-wide association studies (GWAS) have also associated thousands of genetic variants with complex traits, GWAS tends to identify many non-coding, intronic, or intergenic variants which are difficult to interpret (3). This problem is due to linkage disequilibrium (LD) between causal and non-causal variants, which masks the effects of causal variants on the phenotype of interest (4). TWAS mitigates this interpretation issue by prioritizing potential causal genes in addition to genetic variants (4,5).

More than a dozen TWAS software packages have been developed in recent years, including PrediXcan (1), TWAS-FUSION (6), UTMOST (7), FOCUS (8), MR-JTI (9), TIGAR (10), moPMR-Egger (11), kTWAS (12) TisCoMM (13) and others (14–18). Many developments in TWAS have focused on improving GReX imputation accuracy to better identify the genetic component of phenotypic variation. Current methods typically use linear models, such as the ElasticNet variable selection model in PrediXcan and the Bayesian sparse linear mixed model (BSLMM) in TWAS-FUSION. To mitigate the low sample size of many tissues available in reference panels like GTEx (2), UTMOST (7) combines multiple single-tissue association scores into a more powerful joint-tissue test to quantify overall gene-disease association. PrediXcan, TWAS-FUSION and UTMOST are currently the three most popular TWAS tools by citation count. Biologists have used these emerging TWAS software packages to identify and interpret causal genes in multiple diseases and domains, such as calcific aortic valve stenosis (19), high-grade serous ovarian cancer (20), breast cancer (21), macular degeneration (22) and schizophrenia (23–26). Several TWAS software packages have also been modified to utilize summary statistics (6,10,25), allowing biologists to analyze an increasing number of publicly available summary-level GWAS datasets (e.g. dbGaP).

Although TWAS has successfully been applied to discover causal genes in multiple diseases, several limitations prevent TWAS from achieving the popularity of GWAS. First, there is currently no resource providing a comprehensive listing of TWAS-discovered gene-disease associations based on published GWAS summary statistics. While many resources are available for recording GWAS significant signals and variants (e.g. CAUSALdb (27), GWAS Catalog (28), GWASdb (29), GWAS Atlas (30) and GRASP (31)), only one such resource is available for TWAS findings (TWAS-hub, http://twas-hub.org/). However, TWAS-hub has significant limitations, as it only implements one TWAS software package (TWAS-FUSION), only contains 342 disease/non-disease traits, and has not been updated since September 2018. Second, the performance of TWAS depends critically on choosing an appropriate causal (disease-relevant) tissue as a reference panel, as GReX is highly tissue-specific for a given disease (32). Many current TWAS studies have arbitrarily selected a reference tissue such as ‘Whole Blood’, which limits their statistical power. Third, the complexity of TWAS software pipelines poses a significant barrier to biologists looking to conduct their own TWAS analyses.

The webTWAS database was developed to address aforementioned three issues. webTWAS applies the three most common TWAS methods (PrediXcan/S-PrediXcan, TWAS-FUSION, and UTMOST) to a curated collection containing the majority of published disease GWAS datasets with complete summary statistics. The statistical models in webTWAS are drawn from three software packages: the Elastic Net and Mashr models are from the PrediXcan/S-PrediXcan software package; the BLUP, Lasso, best-TWAS and Top1 models are from the TWAS-FUSION software package, and the joint tissue GBJ model is from the UTMOST software package. A convenient web interface allows users to explore gene- and disease-level disease association statistics across multiple studies, and search for related diseases using the integrated MeSH ontology tree (33). Users can conveniently download the curated GWAS summary statistics as well as any search results found on webTWAS. To address the disease-associated tissue specificity problem, tissue-specific enrichment analysis (32,34) is used to prioritize reference panels from the top 1–3 most relevant tissues for a given disease. Moreover, to improve the convenience and accessibility of TWAS to biologists, webTWAS provides a web server application for promptly conducting custom TWAS analyses on user-uploaded GWAS summary statistics. webTWAS is an open access resource which is freely available at http://www.webtwas.net/.

## MATERIALS AND METHODS

### GWAS curation and ontology mapping

A repository of 1298 high-quality disease GWAS summary statistics is used to conduct TWAS analyses. The process for curating GWAS data follows that of our previously published resource CAUSALdb (27). Two categories of publicly available GWAS summary statistics are collected based on whether the cohort under investigation is from UKBB or non-UKBB. UKBB cohort data is collected from Neale Lab UKBB v3 (http://www.nealelab.is/uk-biobank), Gene ATLAS (35) and GWAS ATLAS (30). Although these sources are all derived from UKBB, their summary statistics may vary due to differences in sample selection, quality control processes, and the type of association model used. Non-UKBB cohorts include GWAS summary statistics from public databases such as GWAS Catalog (28), LD Hub (36), GRASP (31), PhenoScanner (37) and dbGaP (38), as well as summary statistics curated from consortium websites such as PGC (https://www.med.unc.edu/pgc), MAGIC (https://www.magicinvestigators.org), SSGAC (https://www.thessgac.org) and JENGER (http://jenger.riken.jp/en/).

Duplicate summary statistics from multiple publication sources are removed by retaining only the source with the most information available. Sources are included only if information regarding sample size, population, and the original publication can be extracted. Population information is mapped to the five super-populations (AFR, AMR, EAS, EUR and SAS) from the 1000 Genomes Project (1KGP) (39). The GTEx reference panel used by webTWAS consists mainly of individuals of European ancestry (40,41), and is not suitable for imputing GReX in non-European individuals due to variations in gene expression between different populations. We therefore only include GWAS statistics from the European super-population (EUR) in the current version of webTWAS. The diseases reported by each GWAS are manually mapped to Medical Subject Headings (MeSH) (33). To ensure accurate trait mapping, we include MeSH labels based on additional information from the data source, original publications related to the source, and related terms from the MeSH website (https://meshb.nlm.nih.gov/search). For traits reported in the UKBB cohort, descriptions from ICD10 (https://icd.who.int/browse10/2016/en) and related notes found on UKBB Showcase are also included as MeSH labels.

### GWAS quality control

Summary-statistic based TWAS software packages usually require several association statistics to be included for each variant (such as variant coordinate, dbSNP ID, effect/non-effect allele, *P*-value, beta coefficient and *Z*-score). To ensure that curated GWAS datasets match the input requirements of these TWAS software packages, we performed several quality control steps on the raw GWAS data. First, we inspect the coordinates and dbSNP ID (rsID) of each variant. If the rsID is missing, we extract it from dbSNP build 151 using the variant coordinates. Variants are excluded if the coordinates and rsID are both missing. Second, summary statistics must explicitly define both effect and non-effect alleles. When only the effect allele is available, the non-effect allele is inferred from biallelic sites in 1KGP. Variants are excluded if the non-effect allele cannot be clearly determined. Third, we discard summary statistics that do not have both a *P*-value and beta coefficient, as a *Z*-score can be calculated from the *P*-value and beta coefficient.

### TWAS analysis models

To identify causal genes and variants, webTWAS uses seven different statistical models based on three popular and representative TWAS software packages: PrediXcan/S-PrediXcan (ElasticNet and Mashr models), TWAS-FUSION (best-TWAS, BLUP, LASSO, and Top1 models) and UTMOST (joint tissue GBJ model). S-PrediXcan is an extension of PrediXcan which allows PrediXcan's results to be computed from summary statistics (25). Although the Top1 model is underpowered according to previous studies (5,42), we have retained the Top1 model as it is integrated in the TWAS-FUSION software package. To remind users of this issue, we highlight the Top1 model with the label ‘The Top1 model is underpowered according to previous studies, and is included in webTWAS for reference purposes’.

For each gene, each of the models is fit separately to each of the 47 GTEx tissues as reference panels. A total of 47 × 6 + 1 association results are computed from these 47 × 7 model-tissue pairs, as the joint tissue GBJ model from UTMOST combines the results of all tissues into a single score. The GReX imputed by each of these model-tissue pairs is then used to identify potential causal genes from GWAS summary statistics. We apply the default LD matrix for SNPs provided by each TWAS software package. In particular, the LD matrix for PrediXcan/S-PrediXcan, TWAS-FUSION and UTMOST are all drawn from the 1000 Genomes Project. The Bonferroni-corrected significance level is implemented as $\frac{0.05}{nx}$ to account for multiple testing (43), where $nx$ is the total number of genes. We use default parameters for all TWAS software packages.

### Disease-specific tissues

Although many studies (13,25,32,34,44,45) have shown that gene expression is highly tissue dependent, an arbitrary reference tissue is often used in many TWAS analyses when the causal tissue type is unknown. To address this issue, webTWAS uses the methodology from TSEA-DB (34) to identify trait-specific tissues for a given target disease. First, gene-based *P*-values are calculated by the Pathway scoring algorithm (Pascal) (46). Disease-associated gene (TAG) sets are defined as the genes with P-values less than a cutoff threshold set to 0.05 (note that TSEA-DB also includes TAG sets with thresholds of 0.01, 0.001 and 0.0001). For each TAG set, the chi-square association test from deTS (32) is used to select up to three tissues which are the most significant to the target disease. TAG samples with fewer than 20 or more than 3000 genes are excluded as they are not suitable for analysis by deTS. Using this tissue-specific enrichment analysis pipeline, at least one disease relevant tissue was identified for all but 45 of the GWAS summary statistics datasets.

### Web server for online TWAS analysis

In addition to listing precomputed associations, webTWAS also includes a web server for users to conduct custom TWAS analyses. This feature requires users to upload a GWAS summary statistics file containing columns for SNP rsID, effect allele, non-effect allele, and either *P*-values or *Z*-scores. Users can select any of the 47 GTEx tissues as reference panels. To select an appropriate disease relevant tissue, we recommend applying deTS as described previously, or using pre-computed trait-specific tissues from resources such as TSEA-DB. Users can run six different statistical models based on two popular and representative TWAS software packages: PrediXcan/S-PrediXcan (ElasticNet and Mashr models) or FUSION (best-TWAS, BLUP, LASSO and Top1 models), and modify the default *P*-value cutoff as needed.

### Database and webserver structure

**Figure 1.**
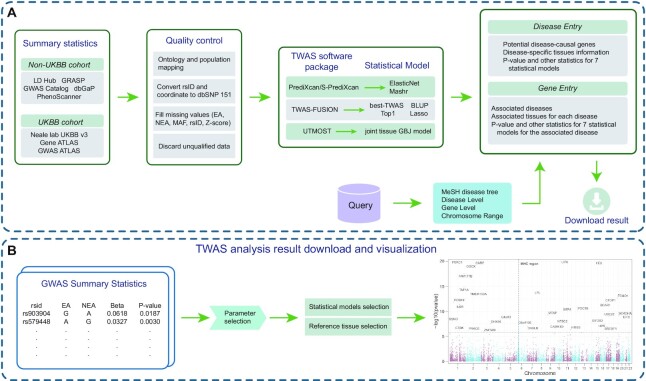
The overall architecture of the webTWAS platform. (**A**) Data processing workflow and query results. (**B**) Framework of online web server for running custom TWAS analyses.

The back-end of webTWAS is developed in the Java-based Spring Boot web framework. The front-end is developed with the VueJs framework, and the user interface uses the Element UI framework for VueJS. A MySQL database is used to rapidly retrieve curated GWAS summary statistics and TWAS-identified disease potential causal genes. The web server for conducting custom TWAS analyses uses an asynchronous design to ensure efficient scheduling of job processes. Job processes are recorded and tracked in the webTWAS user interface. The overall architecture of webTWAS is shown in Figure 1.

## RESULTS

### webTWAS statistics and ontology mapping results

We began by collecting disease GWAS summary statistics for European super-populations across various resources and publications (details in Materials and Methods). As of the latest update to webTWAS in July 2021, this process has curated 1298 high quality GWAS summary statistics in total, of which 864 belong to the UKBB cohort and 434 belong to non-UKBB cohorts.

A total of 235 064 pairs of disease/reference tissue and potential causal gene associations are stored in webTWAS (disease-gene pairings are considered distinct if identified using different reference tissues). Among the 24 782 genes with at least one disease association, the average number of associations per gene is 9.49, while the average number of reference tissues in which the gene is identified as significant by any of the applied TWAS models is 5.20.

The 1298 diseases identified in the GWAS datasets in webTWAS were mapped to 887 Medical Subject Headings (MeSH) terms (one or more diseases can map to one or more MeSH terms). We manually mapped reported traits from each dataset to MeSH, accounting for some auxiliary information from the original studies and other descriptions (details in Materials and Methods). webTWAS uses the same tree structure as the MeSH browser (https://meshb.nlm.nih.gov/treeView) to display reported diseases.

### Database usage and interface

**Figure 2.**
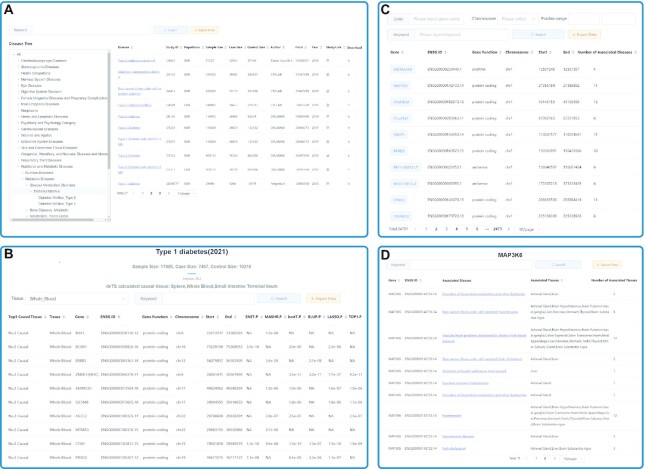
Interface of webTWAS resource. (**A**) Interface for searching GWAS datasets/publications by disease and by browsing the MeSH ontology tree. (**B**) Example of disease webpage, showing potential causal genes for type 1 diabetes. (**C**) Interface for searching TWAS associations by gene. (**D**) Example of gene webpage, showing associated diseases for gene MAP3K6.

Users can search for results stored in webTWAS by querying a disease, gene or chromosome region (Figure 2). If searching for GWAS datasets by disease, users can query by disease name or use the MeSH tree to explore related categories of diseases (Figure 2A). GWAS summary statistics and their originating studies are listed with their disease names, sample sizes, population, number of cases/controls, number of variants with summary statistics, publication information, source links, and mapped MeSH terms. We also provide a download link for each curated GWAS summary statistic. The downloaded statistics include variant coordinates, dbSNP IDs, effect/non-effect alleles, *P*-values, beta-coefficients (BETA) and *Z*-scores, as well as minor allele frequencies (MAF) and standard errors (SE) if available from the original source.

TWAS analysis results are presented with each disease/reference tissue and causal gene pair listed separately, including information such as the top 3 trait-specific tissues identified by deTS, the potential casual gene, the reference tissue used, and trait association statistics for each of the seven statistical models implemented in webTWAS. For the trait association statistics in particular, the *P*-value, effect size, $R^2$, and *Z*-score are provided for the Elastic Net model from PrediXcan/S-PrediXcan, while the *P*-value, effect size, and *Z*-score are provided for the Mashr model of PrediXcan/S-PrediXcan ($R^2$ is the GReX model's coefficient of determination, or the proportion of variance in tissue gene expression accounted for by the model). The *P*-value, $R^2$ and *Z*-score are provided for the four models from TWAS-FUSION, while the *P*-value is the only available statistic available from UTMOST. A drop-down list of tissues is provided on each disease webpage to allow users to filter results by their tissue of interest.

The search interface of webTWAS utilizes the Elasticsearch search engine. If searching for causal genes by gene or genomic location, users can query by gene name, ENSG ID, or chromosome region (Figure 2C). Users can also search the contents of any column (such as publication year or PMID). Complete search results can be downloaded for further analysis by clicking the ‘Export Data’ button.

### Online TWAS analysis

**Figure 3.**
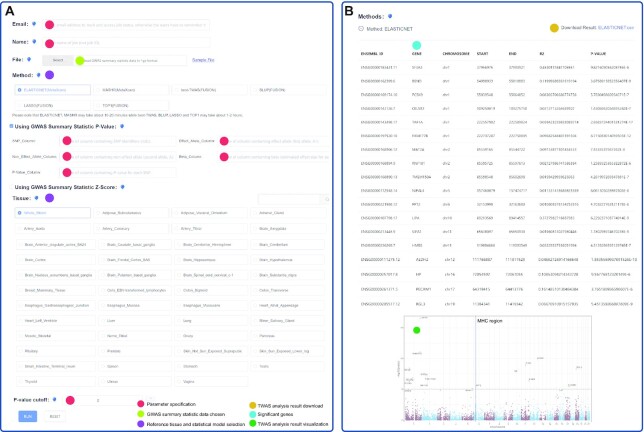
Interface for online TWAS analysis. (**A**) Job submission interface. Users must specify parameters (red), upload GWAS summary statistics (light green) and select a reference tissue/statistical model (purple). (**B**) Example of job results webpage showing table of significant genes (blue), link to download CSV file (yellow) and Manhattan plot (dark green).

The online TWAS analysis component of webTWAS is a web-based server implementation of two popular TWAS software packages (PrediXcan/S-PrediXcan and TWAS-FUSION) for running TWAS analyses and identifying significant disease-associated genes. This web server has an easy-to-use interface which is freely accessible, does not require logins, and enables users to conduct highly customizable TWAS analyses without requiring bioinformatics skills or prior experience with TWAS software. The online analysis consists of four steps (Figure 3): (i) Uploading GWAS summary statistics data. (ii) Specifying parameters such as email address (optional), job name, and *P*-value cutoff (default is 0.05). (iii) Selecting the reference tissue (default is ‘Whole Blood’, the most common reference tissue) and statistical model type (default is the Elastic Net model from PrediXcan, a common and fast-running tool). (iv) Visualizing TWAS analysis results. Results can be downloaded as a comma separated value (CSV) text file, and as a Manhattan plot which is automatically generated by webTWAS to visualize significant genes. Upon job submission, user will receive email notifications when a job starts or ends. Users can also retrieve TWAS analysis results using the job ID provided by webTWAS.

### Comparison to TWAS-hub

Compared with TWAS-hub, which is the only currently available TWAS resource, webTWAS is a more comprehensive resource with multiple advantages: (i) TWAS-HUB only has 342 traits (including non-disease traits such as ‘smoking status’), whereas webTWAS has curated 1298 disease GWAS datasets. (ii) TWAS-hub was last updated in September 2018, whereas webTWAS will be updated bimonthly from July 2021 onwards. (iii) The number of gene-trait associations in TWAS-hub is 75 951, while webTWAS has 235 064 gene-trait associations. (iv) TWAS-hub only implements TWAS-FUSION, while webTWAS implements three TWAS software packages (PrediXcan/S-PrediXcan, TWAS-FUSION and UTMOST). (v) webTWAS uses tissue-specific enrichment analysis (deTS) to determine which tissues are most strongly associated with disease. This improves statistical power by accounting for the TWAS tissue specificity issue (where GReX depends strongly on the reference tissue). (vi) webTWAS presents the first online platform for users to run custom TWAS analyses.

## DISCUSSION

TWAS is a powerful technique which is robust to the linkage disequilibrium and context-dependent regulatory mechanisms that prevent GWAS from accurately detecting causal genes. However, the performance of TWAS is also limited by factors such as gene co-expression, tissue selection bias, and eQTL loci sharing by adjunct genes. Many publications have sought to address these limitations by imputing GReX for trans-eQTLs, imputing cross-tissue gene expression, and integrating kernel machines into the calculation of trait associations, among other methods. As each approach has different performance and benefits depending on the genetic structure, a method is needed to integrate results from multiple TWAS software packages.

The development of a single gene confidence score to integrate results from different TWAS models is a challenging and important problem. We investigated a potential approach where each model is weighted by its accuracy relative to the DisGeNET gene-disease score (47) as a gold standard. DisGeNET is a popular GWAS resource which collects genes and variants associated to human diseases, and the DisGeNET score reflects the strength of a particular gene-disease association based on current knowledge (47). However, the DisGeNET score is an in-house developed metric (47), and may not be a robust basis for estimating the accuracy of TWAS statistical models. Thus, the current version of webTWAS does not include a DisGeNET-like gene confidence score for summarizing the results of the provided TWAS models. We will continue to explore methods for calculating such a score in the future.

Although TWAS has enjoyed substantial research attention and many TWAS software packages have been developed, the complexity of TWAS analysis pipelines is a significant barrier to their use. In particular, no online tool for conducting TWAS analyses is currently available. webTWAS provides a user-friendly online TWAS analysis platform, which allows biologists to conveniently run six statistical models from two TWAS software packages and visualize the results in a Manhattan plot.

In addition, TWAS software packages also lack best practices for users to follow. We propose that the design of the webTWAS database pipeline and web server serves as a basic set of best practices for conducting TWAS analyses. However, more work is needed to improve the completeness and reliability of webTWAS. Our future work will involve integrating new and existing popular summary statistics-based TWAS methods into the webTWAS pipeline, as well as designing a DisGeNET-like gene confidence score to better prioritize true causal genes. Any additional TWAS software packages included into the database will also be integrated with our web server to provide a standardized platform for conducting the latest TWAS analyses using best practices. Finally, newly available GWAS summary statistics will be curated bimonthly for analysis by the webTWAS pipeline, in order to maintain webTWAS as an up-to-date resource for TWAS-identified causal genes.

## DATA AVAILABILITY

PrediXcan, https://github.com/hakyim/PrediXcan/
TWAS-FUSION, http://gusevlab.org/projects/fusion/
UTMOST, https://github.com/Joker-Jerome/UTMOST/
deTS, https://github.com/bsml320/deTS/
PASCAL, https://www2.unil.ch/cbg/index.php?title=Pascal/
1000 Genomes Project, https://www.internationalgenome.org/
GTEx, https://gtexportal.org/
